# Effects of ECT in treatment of depression: study protocol for a prospective neuroradiological study of acute and longitudinal effects on brain structure and function

**DOI:** 10.1186/s12888-015-0477-y

**Published:** 2015-05-01

**Authors:** Leif Oltedal, Ute Kessler, Lars Ersland, Renate Grüner, Ole A Andreassen, Jan Haavik, Per Ivar Hoff, Åsa Hammar, Anders M Dale, Kenneth Hugdahl, Ketil J Oedegaard

**Affiliations:** 1 Department of Radiology, Haukeland University Hospital, Bergen, Norway; 2 Department of Clinical Medicine, University of Bergen, Bergen, Norway; 3 Division of Psychiatry, Haukeland University Hospital, Bergen, Norway; 4 Department of Clinical Engineering, Haukeland University Hospital, Bergen, Norway; 5 NORMENT, KG Jebsen Centre, Oslo University Hospital and University of Oslo, Oslo, Norway; 6 K.G. Jebsen Centre for Neuropsychiatric Disorders, Department of Biomedicine, University of Bergen, Bergen, Norway; 7 Department of Heart Disease, Haukeland University Hospital, Bergen, Norway; 8 Department of Biological and Medical Psychology, University of Bergen, Bergen, Norway; 9 Departments of Neurosciences, Radiology and Psychiatry, University of California, San Diego, CA USA; 10 Multimodal Imaging Laboratory, University of California, San Diego, CA USA

**Keywords:** Electroconvulsive therapy, Magnetic Resonance Imaging, Depression

## Abstract

**Background:**

Major depression can be a serious and debilitating condition. For some patients in a treatment resistant depressive episode, electroconvulsive treatment (ECT) is the only treatment that is effective. Although ECT has shown efficacy in randomized controlled trials, the treatment is still controversial and stigmatized. This can in part be attributed to our lack of knowledge of the mechanisms of action. Some reports also suggest potential harmful effects of ECT treatment and memory related side effects have been documented.

**Methods/design:**

The present study will apply state of the art radiology through advanced magnetic resonance imaging (MRI) techniques to investigate structural and functional brain effects of ECT. As a multi-disciplinary collaboration, imaging findings will be correlated to psychiatric response parameters, neuropsychological functioning as well as neurochemical and genetic biomarkers that can elucidate the underlying mechanisms. The aim is to document both treatment effects and potential harmful effects of ECT.

Sample: n = 40 patients in a major depressive episode (bipolar and major depressive disorder). Two control groups with n = 15 in each group: age and gender matched healthy volunteers not receiving ECT and patients undergoing electrical cardioversion (ECV) for atrial fibrillation (AF). Observation time: six months.

**Discussion:**

The study will contribute to our understanding of the pathophysiology of major depression as well as mechanisms of action for the most effective treatment for the disorder; ECT.

## Background

### Bipolar and major depressive disorder

Bipolar disorder and major depressive disorder (MDD) are mental disorders with a 12-month prevalence in the EU of about 1 and 7%, respectively [1]. They are associated with a reduced quality of life, an increased mortality risk, and are a major cause of inability to work [2,3].

The management of depression includes psychosocial treatment approaches, pharmacotherapy and, for the most severe and treatment resistant patients, electroconvulsive treatment (ECT).

### Electroconvulsive treatment

The idea that convulsions could treat mental illness can be traced to the 16^th^ century, when camphor oil was used to induce convulsions. Seizure-induction by application of electrical current to the human brain was introduced by the Italians Cerletti and Bini in 1938 [4]. Since its introduction, ECT has been applied to various psychiatric and some somatic conditions. Modern ECT has fewer indications and has been developed with the aim to reduce side effects [5].

For some patients in a treatment resistant depressive episode, ECT is the only treatment that is effective. ECT is generally considered to be safe and has shown efficacy in randomized controlled trials [6]. A recent randomized controlled trial found ECT to be more effective than pharmacological treatment for treatment-resistant bipolar depression [7]. However, the treatment is still controversial and stigmatized [8]. This can in part be attributed to our lack of knowledge, since the mechanisms of action is still largely unknown.

Some have compared ECT to lobotomy [9] or hypothesize that ECT affects the brain in a manner similar to severe stress or trauma [10]. Others regard it as a safe treatment that is underused [11], and a systematic review found no persistent cognitive deficits after ECT [12]. A recent randomized controlled trial of right unilateral ECT in treatment resistant depression found no changes in general neurocognitive function, but reduced autobiographical memory consistency after ECT [13]. This finding is in line with subjective patient reports [14], and further research is required.

The NICE guidelines states: “Consider ECT for acute treatment of severe depression that is life-threatening and when a rapid response is required, or when other treatments have failed” [15]. This is in line with the Norwegian national guidelines that recommend ECT in major depression when other treatments have been ineffective (Evidence level A, [16]), and there has been an increase in its use in recent years [17].

Increased knowledge gained through thorough scientific investigations can reduce stigmata and inform patients and health care providers to make appropriate use of ECT. Better understanding of ECT and its mechanisms of action may help patients to cope with side effects and contribute to the development of new treatment options.

### Possible mechanisms of action of ECT

More than one hundred theories have been suggested for the effects of ECT [5]. Although changes to brain structure in major depression have been confirmed by several meta-analysis [18-20] and ECT-induced structural and functional changes have been characterized (for recent reviews see [21-23]) we still lack a unifying theory for its mechanisms of action. The project will focus on three suggested effects of ECT, each reflecting proposed pathophysiological changes and mechanisms of action, see below. As a multidisciplinary study, results from the neuroradiological measures can be correlated to biomarkers in blood and behavioral parameters; e.g. improvement/remission after ECT should be correlated to improved performance on neuropsychological testing. For dichotic listening, improved scores in the forced left condition would indicate better cognitive control.

### Hippocampal volume

The human nervous system adapts to challenges. It can be changed by learning as well as by pathological conditions, such as psychiatric disorders. One structure that has been studied in large detail in this regard is the hippocampus; a structure that is important for learning and memory. Hippocampal volumes are *reduced* in major depressive disorder [19,24,25] and in a number of other psychiatric and somatic disorders (reviewed in [26]). The volume reduction of the hippocampus has been associated with duration of untreated depression [27]. On the other hand, *increased* hippocampal volumes can occur after extensive learning, e.g. studying to become one of London’s taxi drivers [28,29]. The *increase* in hippocampal volumes may be related to neurogenesis, which has been shown to occur in animal models [30,31]. In primates, the proliferation of granular cells in the dentate gyrus of the hippocampus was shown to be reduced by stress [30]. Seizures induce neurogenesis in rodents [32], and animal models have shown electroconvulsive seizures to have effects on neurotransmitters, gene expression, growth factors (such as Brain derived neurotrophic factor - BDNF, Vascular endothelial growth factor - VEGF, Fibroblast Growth Factor - FGF) and neuropeptides (such as neuropeptide Y - NPY, Thyrotropin-releasing hormone - TRH, VGF) and lead to synaptic remodeling and cellular proliferation (reviewed in [33]). Research from animal models also indicate that electroconvulsive shocks can reverse the effect of cortisol and even cause an increase of hippocampal volumes (reviewed in [5]).

Increased levels of BDNF has been reported following ECT [34], and BDNF has been suggested as a potential biomarker for depression [35]. Neurogenesis has been shown to occur in the dentate gyrus of the hippocampus in adult humans [36], and ECT-induced neuroplasticity is gaining more focus as a framework for understanding the effects of ECT [23]. A few studies of humans have reported increased hippocampal volumes and/or other structural changes following ECT [37-41]. Nordanskog et al. [39,41] performed manual segmentation without complete blinding of the MRI time point that was traced (before or after ECT), introducing a potential observer bias. Dukart et al. [38] used voxel-based morphometry, while Tendolkar et al. [40] and Abbot et al. [37] both used FreeSurfer [42] for volumetric segmentation and analysis. Compared with these studies we will recruit more patients, use state of the art automatic segmentation procedures, and radiology readers will be blinded to study group and the time point of MRI scans. In addition, by applying multimodal imaging, structural changes can be assessed with respect to changes in diffusion properties, susceptibility weighted imaging (SWI) and fluid attenuated inversion recovery (FLAIR) imaging. Our design will allow longitudinal tracking of brain changes; 1-2 hours after the first ECT, after treatment completion and at 6 months follow up.

### Gamma-Aminobutyric acid (GABA) and connectivity

ECT has anticonvulsive effects and is sometimes used in the treatment of status epilepticus [43]. One hypothesis suggests that the magnitude of increase in seizure threshold, induced by ECT, is important for the antidepressant efficacy [44]. Drugs that enhance GABAergic neurotransmission are known for their anticonvulsant effect, and the role of amino acid neurotransmission systems, particularly reduced function of GABAergic neurotransmission has been increasingly appreciated in major depression (for reviews, see [45,46]). Tiagabine, a selective GABA reuptake inhibitor was shown to be effective in treatment of depression with anxiety [47]. A post mortem study of gene expression in elderly depressed patients found alterations in GABA and glutamate pathways markers indicating diminished activity in the anterior cingulate cortex (ACC) [48]. A recent meta-analysis suggested increased resting-state activity in the rostral ACC as a biomarker for treatment response in major depression, and a shift from GABA- to glutamate-mediated modulation was suggested [49].

Interestingly, one early study found increased concentrations of cortical GABA after ECT in depressed patients by use of proton magnetic resonance spectroscopy (^1^H-MRS) [50], however this finding has to our knowledge not been reproduced by other groups.

It has recently been suggested that “hyperconnectivity” in networks involved in mood regulation can be reduced after a course of ECT [51,52]. This finding may seem contrary to findings of increased fractional anisotropy (FA) in frontal limbic projections after a course of ECT [53,54]. FA is often regarded as a measure of white matter tract integrity and increased axonal integrity may seem contra-intuitive if one expects reduced connectivity after ECT. Possible explanations could be that the projections that are “enhanced” by ECT are GABAergic, or that improved integrity of certain projections may lead to more coordinated electrical activity in these projections, which overall is detected as “reduced connectivity”.

Our project will encompass measures of neurotransmitters (GABA and glutamate by ^1^H-MRS) and diffusion parameters (e.g. fractional anisotropy, mean diffusivity and separation of restricted and hindered water by Restriction Spectrum Imaging, RSI [55]), enabling longitudinal investigations of GABA- and glutamate levels as well as white matter properties in the same patients.

### ECT and harmful effects

ECT has been a controversial treatment from its introduction. The most important side effects are related to memory impairments [6] and it is recommended that the patients’ cognitive functioning is monitored both during and after treatment [16]. A recent randomized controlled trial in treatment-resistant bipolar depression found reduced autobiographic memory consistency after ECT but no deterioration of general neurocognitive function [13]. Structural damage to the human brain has to our knowledge, never been documented to be caused by ECT. Case studies with rare complications, such as subdural hematoma, have been published [56], however a study using cerebral Computer Tomography in 40 patients before and after ECT detected no changes caused by ECT, even with convulsions lasting several minutes [57]. Both conventional MRI and diffusion weighted imaging (DWI; a sequence that is sensitive to edema) have failed to find structural damage [58]. However, changes on DWI have been shown for patients after status epilepticus [59]. If brain injury occurs as a consequence of ECT, one may expect to find micro hemorrhages. SWI is extremely sensitive to hemorrhages, and is routinely used in imaging of stroke [60]. However, SWI, as an indicator of microvascular dis-integrity, has to our knowledge never been applied after ECT. In addition, by using ^1^H-MRS we will measure N-acetylaspartate (NAA), which is primarily localized in neurons and considered a marker for neuronal integrity [61].

Our project will use high field strength, state of the art MRI and combine RSI, SWI and ^1^H-MRS which should enable detection of more subtle post-ECT effects.

### Hypotheses and aims

Based on the above discussions, the following main hypotheses define the outline and aims of this project:

1. Hippocampal volumes increase after ECT treatment. A) Specifically there is increased volume of the dentate gyrus, which would suggest that the increase is caused by neurogenesis. B) Changes in hippocampal structure correlate with treatment response, neurocognitive measures and increased concentrations of neurotrophic factors in blood samples.
2. ECT causes increased levels of the neurotransmitter GABA and changes the glutamate/GABA balance. A) GABA concentrations correlate with treatment response. B) Genes regulating GABA synthesis and cycling are up-regulated or activated. C) A subset of cortical projections is strengthened; a possible mechanism causing reduced connectivity in frontal areas.
3. ECT does not cause measurable signs of harmful effects to the brain. A) No changes are detected on microvascular (SWI) and microstructural (RSI) imaging. NAA (measurend in the ACC) is unaffected. B) Possible immediate post-ECT effects, e.g. edema, that is detectable by diffusion weighted imaging, are reversible.

### Methods and design

The study is prospective and observational, and all patients will receive the standard ECT treatment, as it is provided at the ECT-department at the Haukeland University Hospital.

**Figure 1:**
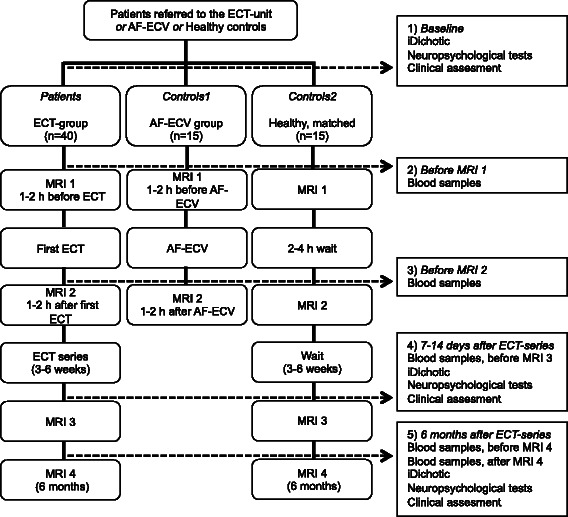
Flow Chart of Study Design.

A flow chart of the study design is shown in Figure 1, and details on study measures and variables are listed in Table 1.

**Table 1:** **Variable overview**

	Study visit	1	2	3	4	5
	Time point test	<7 d of first ECT	1– 2 h before first ECT	1– 2 h after first ECT	7 – 14 d after last ECT	6 mo after ECT-series
	Informed consent	X				
Diagnostic interview	MINI plus	X				
Clinical assessment	Clinical examination	X				
	Illness history, previous episodes and ECT-treatment	X				
	Current and concomitant medication	X			X	X
Symptom severity	MADRS	X			X	X
	CGI	X			X	X
Relapse	Interview					X
Overall cognitive function	MMS	X			X	X
Neuropsychological assessment	WASI	X				X
	CVLT-II, Rey, Digit span from WASI-R, WCST, D-kefs: Color-Word interference test, Word fluency, Tower, TMT, CPT, Digit symbol WAIS-R, Pegboard	X			X	X
Autobiographical memory	AMI-SF	X			X	X
Everyday memory	EMQ	X			X	X
Cognitive control	Dichotic listening	X			X	X
Blood samples	Full blood, EDTA and PAX-gen for biobank		X^1)^	X^1)^	X^1)^	X^2)^
Radiology	MRI caput; T1 FSPGR, T2 CUBE FLAIR, RSI, SWI, MRS, MEGAPRESS		X	X	X	X

^1)^Before MRI.

^2)^Before and after MRI.

Relevant patients with depression are addressed in order to establish whether they are willing to be screened for the study. The patients must be assigned a patient number and sign the consent form after receiving oral and written information about the study prior to undergoing any study procedures.

### Patients

Forty patients accepted for ECT at Haukeland University Hospital will be included. After inclusion of 8 patients, the protocol was slightly revised and the remaining 32 patients will follow the protocol as described here.

### Inclusion criteria

Patients (age > 18) referred to the ECT-unit and accepted for treatment because of moderate and severe depression, fulfilling the criteria for the following ICD-10 diagnoses: F31.3 and F31.4; F32.1 and F32.2 and F32.3; F33.1 and F33.2 and F33.3. In addition the symptom intensity must be verified by a score ≥ 25 on the Montgomery and Åsberg Depression Rating Scale (MADRS). There is no upper age for participation; however, the responsible clinician will consider if patients are eligible for inclusion (functioning, enable to give written informed consent).

### Exclusion criteria

ECT treatment within the last 12 months. Pregnancy. Patients unable to give written informed consent (according to the responsible clinician or ECT responsible). Patients who cannot participate in the MRI scanning because of contraindications to MRI.

### Control groups

There will be two control groups; a group of patients undergoing ECV for AF (*controls 1*) and healthy controls undergoing the same investigations as the ECT patient group, but not receiving ECT or anesthesia (*controls 2*).

### Controls 1

In order to control for the potential effect of anesthesia on MRI images (particularly with regard to the spectroscopy) and blood samples, 15 patients referred for ECV of AF will be recruited. This is a patient group that receives similar anesthesia to ECT patients. This control group will have 2 MRI scans; one 1-2 hours before ECV and another MRI 1-2 hours after ECV. Blood samples will also be collected for the biobank at time points indicated in Figure 1. In addition to being a control group, data that is acquired will be used in a pilot investigation of potential effects of ECV of AF, if there are silent emboli to the brain. Such emboli would readily be detected on the diffusion images. Antithrombotic treatment must be Warfarin with an INR value above 2,0 at all measurements for the last 3 weeks prior to DC cardioversion or absolute compliant everyday use of Non-vitamin K antagonist oral anticoagulants (NOACs) for 3 weeks. There will be no changes in the treatment of their AF, patients are only asked to participate in additional examinations (MRI, blood samples) before and after ECV.

### Controls 2

When analyzing longitudinal MRI data, it is important to control for effects on imaging parameters that are a consequence of repeated measurements/time, rather than effects of the treatment. 15 healthy, age and gender-matched volunteers will be recruited for repeated MR imaging, blood samples and neuropsychological testing; following the protocol for the ECT patients (see Figure 1) but with no ECT or anesthesia.

### ECT treatment

ECT will be administered with a Thymatron System IV Somatics Inc. providing brief-pulse, square wave, constant current.

#### Anesthesia

Anesthesia will be obtained with either the short acting anesthetic thiopental or propofol. All patients will be hyperoxygenated with oxygen-enriched air 1 to 2 minutes before and during the initiation of anesthesia to optimize induction of seizures [62,63]. Other medication necessary during anesthesia (e.g. for premedication or termination of prolonged seizure) will be left to decision by the anesthesiologist.

#### Stimulation electrodes placement

Stimulation electrodes will be placed ad modem d’ Elia [64] (Right unilateral electrode placement, RUL), as high dosage ECT with unilateral placement of stimulation electrodes has shown to be as effective as bilateral placement [65,66]. Three sessions per week will be given until remission, with a maximum of 18 sessions.

#### Stimulus

The duration of the stimulus pulse will be set to 0.5 ms. The initial stimulus energy will be determined by an age based method, where the energy (E) is calculated as following [67]: Patient’s age in years × 5 ≅ stimulus charge in mC. The Thymatron delivers a charge of 25.2 to 504 mC in 20 equal steps, set by the % Energy dial. According to the above formula this makes: Patient’s age in years ≅ % Energy. In order to consider gender specific differences in seizure threshold, the % Energy was adapted as following: For male patients: % Energy + 5 to 10%. For female patients: % Energy - 5 to 10%.

#### Seizure adequacy

The adequacy of each seizure will be evaluated by the ECT-clinician based on seizure duration, δ-waves, reorientation time and clinical effect. The treatment should be followed by a comatose state, from which consciousness is gradually regained [68]. If a sufficient seizure was not obtained in one session the patient will be either re-stimulated in the same session or/and stimulus parameter will be adjusted in the next session.

### Clinical assessments and Neuropsychological tests

Clinical assessments and monitoring will be performed largely in accordance with a recently used protocol [69], as detailed below and summarized in Table 1.

Patients will be diagnosed on the basis of a clinical interview supported by information from hospital records. The diagnoses will be subsequently verified by the Mini-International Neuropsychiatric Interview (MINI; specifically the MINI-Plus) [70]. Symptom intensity will be measured with MADRS [71] and the Clinical Global Impression (CGI) [72].

Patients will be assessed before the treatment, and weekly during the ECT-series with the Mini-Mental State (MMS) by their treating clinician.

A neuropsychological test battery that includes both standardized and normalized tests and experimental methods to assess memory, attention, psychomotor speed and executive functions, will be applied at inclusion, after treatment and at follow up, as listed in Table 1. The neuropsychological assessment will consist of standardized tests measuring cognitive functioning within verbal and visual memory with California Verbal Learning Test-II (CVLT-II) Rey Complex Figure Test (RCFT) and Digit span from Wechsler Adult Intelligence Scale, Revised (WAIS-R), executive functioning with Wisconsin Card Sorting Test (WCST), and test from the Delis –Kaplan Executive Function System (D-kefs): Color-Word Interference Test (CWIFT), Verbal fluency (VF), Tower, Trailmaking test (TMT), attention measured with Conners’ Continuous Performance Test-II (CPT), Digit symbol from WAIS-R and motor speed (Pegboard), in addition to general levels of intellectual ability Wechsler’s Abbreviated Scales of Intelligence (WASI). Autobiographical memory will be assessed by using the Autobiographical Memory Interview-Short Form (AMI-SF).

### Dichotic listening task

Placement of electrodes on the non-dominant side (unilateral stimulation) is important to reduce cognitive impairment as a side effect of ECT treatment [65,73]. Traditionally, hemispheric dominance is evaluated by handedness measures, which however is a crude measurement when it comes to subtle differences in function between the cerebral hemispheres. It is therefore suggested to use a neuropsychological task, dichotic listening, which has been shown to be comparable in sensitivity to reveal functional differences between the hemispheres to the Wada test [74,75]. We will apply the Bergen dichotic listening test [76] both as a measure of language dominance and as an effect parameter, i.e. as a measure of cognitive control since it has been shown that varying instructions about attention focus while performing the dichotic listening task reveals capacity for cognitive control [77]. A recent development makes it possible to deliver the test bedside, by use of an application on a hand held device; iDichotic, Bergen fMRI group [78]. The prediction is that treatment response correlates with improved results for the instruction condition that requires highest cognitive control.

### MRI acquisition and post processing

Imaging will be performed at 4 time points: ~1-2 hours before and ~1-2 hours after ECT, ~ 7-14 days after ended treatment and at follow-up 6 months after ended treatment. The same MRI protocol will be applied at each time point (Figure 1).

#### MRI Protocol

Initial imaging will be performed on a 3T GE Signa HDxt system with 8 channel head coil, but most of the subjects will be scanned on a 3T Discovery MR750 system with 32 channel head coil. The protocol (details specified for the MR750 system) includes a T1-weighted fast spoiled gradient echo, FSPGR (TE/TR = 2.9/6.7 ms; TI = 600 ms, flip angel = 8°; FOV = 25.6 cm; voxel size = 1.0 × 1.0 × 1.0 mm^3^, acquisition time = 10:32 min.); a T2-weighted CUBE FLAIR sequence (TE/TR = 129/6000 ms; TI = 1855 ms; FOV = 25.6 cm; voxel size = 1.0 × 1.0 × 1.0 mm^3^, acquisition time = 08:51 min.); for RSI, a single-shot pulsed-field gradient spin-echo EPI sequence (TE/TR = 85/7000 ms; FOV = 24 cm, matrix = 96 × 96 × 55 with 4 b-values (*b* = 0, 500, 1000 and 4000 s/mm^2^ and 6, 6 and 15 unique directions for the nonzero b-values, respectively), acquisition time = 3:30 min.); for SWI a gradient recalled echo 3D Ax SWAN sequence (TE/TR, 23/37 ms; slice thickness 2 mm; acquisition time 3:30 min.). For ^1^H-MRS, both single-voxel point-resolved spectroscopy, SV PRESS, and a spectral editing method, MEGA-PRESS [79], will be used. The SV-PRESS (TR = 1500 ms, TE = 35, 128 scans; acquisition time = 3:48 min.) voxel will measure 2 × 2 × 2 cm^3^ and the placement alternate between right and left anterior cingulate cortex (ACC) for each new patient. For MEGA-PRESS (TR = 1500 ms, TE = 68 ms, 192 scans, acquisition time = 10:06 min.) the voxel will measure 3 × 3 × 3 cm^3^ and cross the mid-line, covering both right and left ACC in every patient.

#### Image processing and analysis

Structural data will be analyzed using FreeSurfer [42] and Quarc [80]. In a preprocessing step, structural images will be corrected for distortions caused by gradient non-linearity [81], diffusion weighted (DW) images will be corrected for motion, eddy currents and magnetic susceptibility artifacts [82,83] and the DW volume will be co-registered to the structural volumes. SWI data will be analyzed using Statistical Parametrical Mapping (SPM8/SPM12) analysis software package (Wellcome Department of Cognitive Neurology) running under MATLAB (Mathworks). RSI data will be analyzed using custom made software and processing and/or with FSL [84,85] and MRS data by using LCModel Software [86]. SPSS will be used for statistical analyses.

Analysis methods and software may change if newer versions or other software is found to be more suitable than the above mentioned.

### Blood samples and biobank

We will analyze multiple peripheral blood biomarkers relevant for the hypotheses outlined in the introduction. Due to the constant progress in the field, the decision on the specific markers to analyze and how to perform the analysis should not be taken too early. However, candidate markers include neurotrophic factors (e.g. BDNF), pro-inflammatory cytokines, neurotransmitter related amino acids, monoamines and related metabolites (e.g. GABA, glutamate, kynurenines, neopterin) and S100B (a marker of damage to the blood-brain barrier). Measurements of peripheral biomarker levels will be supplemented by analyses of DNA variants and peripheral blood mRNA levels (array based genome wide DNA genotyping, methylation profiling of target genes and real-time reverse transcription polymerase chain reaction mRNA measurements). Blood samples (up to 30 ml) will be collected and stored as whole blood, serum and on an RNA stabilization medium at -80°C for later analysis. A dedicated research biobank “Imaging and Depression - ImDep” has been generated for the project using existing infrastructure (storage, alarm and registration) established in a previous project.

### Power analysis

Only one study has measured GABA changes after ECT by MRS in humans, and found an increase from 0.85 (SD = 0.34) to 1.51 (SD = 048) mmol/kg brain tissue, N = 8 [50]. Using a mean difference of 0.6 and a SD of 0.5, α = 0.05 and power of 0.8 the total sample size needed would be 8 (calculated using G*Power 3.1.3, paired t-test, two-tailed).

For analysis of hippocampal volumes, data from Nordanskog et al. [41] were used. For the right hippocampus the mean difference in volumes after a course of ECT was 133 μL with a SD of 123 μL, similar analysis as above indicates that the sample size needs to be at least 9.

We found no studies on SWI and DWI imaging that could be used to estimate power of the suggested study protocol. Based on the analysis above, a total of 10 patients is a minimum. Due to wide inclusion criteria, expected heterogeneity of the sample population and in order to increase the robustness we intend to include 40 patients.

### Ethical considerations

The study is based on written informed consent. Patients will receive standard ECT treatment. The study, and the specific Biobank, are approved by the Regional Committee for Medical and Health Research Ethics, REC South East, Norway.

### For patients and controls

Participation in the project will for the patients include MRI scans (4 time points) and blood samples (5 time points) as well as neuropsychological testing (3 time points) that are not part of the standard treatment regimen. For *controls1* (AF-ECV) participation will include MRI (2 time points) and blood samples (2 time points) that is not part of the standard clinical treatment. For *controls2* participation will require MRI scans (4 time points) and blood samples (5 time points) as well as neuropsychological testing (3 time points). *Controls2* will be economically compensated for participating.

There are no known adverse effects related to MRI scanning when standard safety procedures are followed. However, scanners are noisy and scans will last up to one hour. Blood samples will require venous puncture. A neuroradiologist will screen the first MRI scan for each participant. In the case of incidental findings of relevant pathology, the participant will be offered referral to medical consultation.

### For science and society

As health care professionals we are obliged to ensure that our treatments are well documented and safe. Thorough investigations of ECT effects will improve patients’ feeling of safety and possibly reduce stigmata related to the condition. Depression is a common disorder with substantial costs for individuals, employers, and health and welfare systems. New knowledge about the pathophysiology of major depression as well as mechanisms of action and possible harmful effects of ECT is crucial and may lead to new prospects for future treatments.

## Discussion

By use of multimodal neuroradiological imaging as well as multidisciplinary investigations spanning from genes to behavior, the study aims at increasing knowledge about what ECT does to the brain, such as: a) Does ECT affect microvascular integrity? b) How are hippocampal volumes related to ECT parameters and treatment effects? c) Does GABA-levels increase after ECT?

Several of the measures have to our knowledge never before been applied in this setting, e.g. RSI, SWI, Dichotic listening, and a control group that receives a similar anesthesia and electrical stimulation to the chest. Strengths of the study are comprehensive investigations and a moderately large sample size. Potential weaknesses are the rather broad inclusion criteria and expected heterogeneity in the patients’ use of medications, both of which may increase the variance in study measures. However, ECT is an unspecific treatment and although we expect that its effects on the brain and the human physiology should be largely independent of diagnosis, medication use and age, it will be interesting to see how ECT-induced changes relate to treatment effects and clinical parameters.

### Ethical approval

Regional Committee for Medical and Health Research Ethics, REC South East ID: 2013/1032 ECT and Neuroradiology. Approved first in June 2013, last revision December 2014.
